# DISRUPTING THE NEGATIVE AFFECT AND PAIN CONNECTION IN OLDER ADULTS: THE ROLE OF SOCIAL INTERACTIONS AND ENJOYMENT

**DOI:** 10.1093/geroni/igad104.3285

**Published:** 2023-12-21

**Authors:** Jennifer Graham-Engeland, Dakota Witzel, Erin Harrington, Christopher Engeland, Lynn Martire, Karina Van Bogart

**Affiliations:** Pennsylvania State University, University Park, Pennsylvania, United States; Pennsylvania State University, University Park, Pennsylvania, United States; Pennsylvania State University, University Park, Pennsylvania, United States; Pennsylvania State University, University Park, Pennsylvania, United States; Pennsylvania State University, University Park, Pennsylvania, United States; Pennsylvania State University, University Park, Pennsylvania, United States

## Abstract

Although it is well-established that negative affect is bi-directionally linked with pain, little research has examined their relationship in daily life among older adults. Even less is known about what social factors may help attenuate the affect-pain connection. We examined whether social interactions and reported enjoyment in daily life were associated with an attenuated link between negative affect and two important pain outcomes (pain intensity and perceived interference from pain). This was examined among a socioeconomically and racially diverse sample of 317 older adults aged 70+ (Mage = 77.45 ; 67% women; 40% Black; 13% Hispanic) who were recruited from the Bronx, NY as part of the Einstein Aging Study and who completed ecological momentary assessments five times daily for 14 days. Three-level multilevel models were estimated, controlling for mild cognitive impairment (MCI) status, gender, age, education, body mass index, and average level positive affect for models that did not include enjoyment. In given moments, higher negative affect and lower enjoyment were associated with higher pain intensity and pain interference (ps<.05). Moment-level negative affect and enjoyment significantly interacted to predict both higher pain intensity (p=.005) and pain interference (p<.0001), with patterns suggesting a buffering effect of enjoyment. In addition, a three-way interaction emerged such that during moments when no interactions occurred and negative affect was lower than a person’s average, there was a buffering effect of momentary enjoyment on pain intensity. Findings extend understanding of the affect-pain connection and the potential mitigating impact of social interactions and moments of enjoyment.

